# Indium-Zinc-Tin-Oxide Film Prepared by Reactive Magnetron Sputtering for Electrochromic Applications

**DOI:** 10.3390/ma11112221

**Published:** 2018-11-08

**Authors:** Ke-Ding Li, Po-Wen Chen, Kao-Shuo Chang, Sheng-Chuan Hsu, Der-Jun Jan

**Affiliations:** 1 Department of Materials Science and Engineering, National Cheng Kung University, Tainan 70101, Taiwan; tyty01068@gmail.com (K.-D.L.); kschang@mail.ncku.edu.tw (K.-S.C.); 2 Division of Physics, Institute of Nuclear Energy Research, Taoyuan County 32546, Taiwan; g923319@iner.gov.tw (S.-C.H.); djjan@iner.gov.tw (D.-J.J.)

**Keywords:** indium-zinc-tin-oxide (IZTO) film, electrochromic device (ECD), tungsten electrode film, DC reactive magnetron sputtering, cathodic arc plasma (CAP)

## Abstract

This paper reports on the fabrication of indium-zinc-tin-oxide (IZTO) transparent conductive film deposited by direct current (DC) reactive magnetron sputtering. The electrical, structural, and optical properties of IZTO film were investigated by Hall measurement, X-ray diffraction (XRD), and optical transmission spectroscopy with various sputtering powers. The IZTO film prepared used power at 100 W showed the lowest resistivity of 5.2 × 10^−4^ Ω cm. To accomplish rapid switching and high optical modulation, we have fabricated an electrochromic device (ECD) consisting of an working electrode (WO_3_ electrode film deposited on IZTO/ITO/glass) and a counter-electrode (Pt mesh) in 0.2 M LiClO_4_/PC liquid solution. The device demonstrated an optical contrast of 44% and switching times of 4.6 s and 8.1 s for the coloring and bleaching state, respectively, at the wavelength of 550 nm.

## 1. Introduction

An electrochromic device (ECD) is based on a well-known reversible optical switching phenomenon and is an enabling technology for smart windows, optical information displays, variable-reflectance mirrors, electronic papers, and switch mirrors [1]. Electrochromism can produce an interesting phenomenon as reversible change in optical properties under a small applied DC voltage pulse difference [2]. Smart windows based on electrochromic (EC) materials can be commanded to reversibly change their optical properties of reflectance, transmittance, and absorption. Smart windows can effectively reduce the heating or cooling loads of building energy consumption [3]. Tungsten oxide (WO_3_) electrode film has received extensive attention to inorganic transition metal-oxides due to its attractive cycle life, including environmental stability and stability against sunlight exposure [4]. The chemical reaction of a WO_3_ electrode film are due to reversible oxidation/reduction reactions induced by electrochemical double injection/extraction of positive ions (lithium or proton) and electrons into/outside the host of WO_3_ lattice in transition of W^5+^ and W^6+^ [3,4].

**Figure 1:**
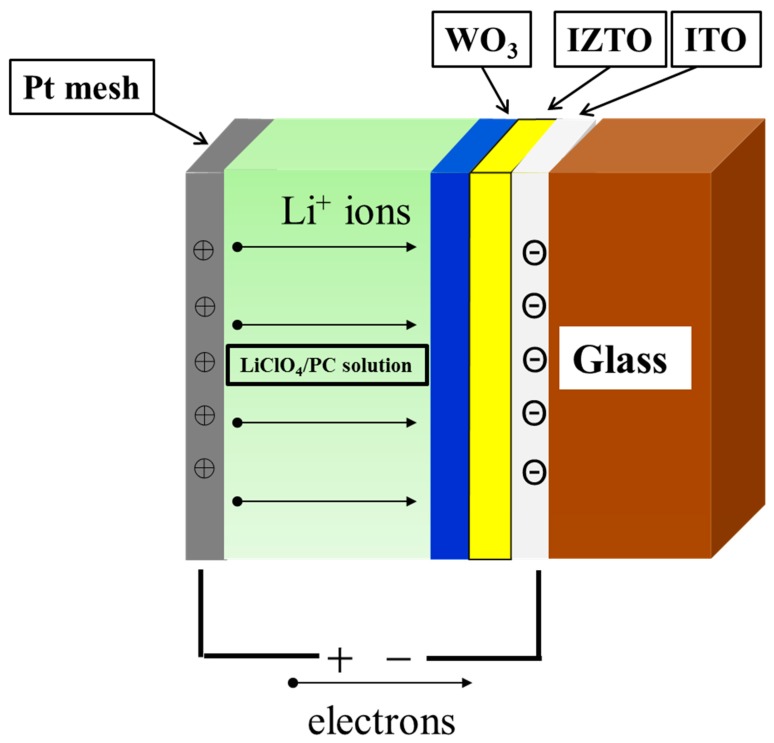
The typical ECD structure.

The typical and promising structure of an ECD is composed of Pt mesh/electrolyte layer/electrochromic layer/transparent conducting electrode layer, as shown in Figure 1. The transparent conducting electrode is the key point studied in this article. However, indium tin oxide (ITO), known for its good electrical conductivity, has to be deposited at high temperatures and then post-annealed at a temperature above 200 °C. This annealing temperature causes the ITO films to be crystallized and roughened [5]. Sun et al. suggested an EC device with a ZnO nanowire array modified surface of ITO has fast-switching response [6]. The nanostructure of a ZnO film provides many direct pathways for fast electron transport and porous ZnO nanowires deposited on surface of WO_3_ film [6,7]. The nanostructure of a WO_3_ electrode film can be an increased active site for redox chemical reaction [8]. Amorphous indium zinc-tin oxide (IZTO) also has good electrical conductivity and a high transmittance in the visible region of the spectra. Amorphous IZTO can be deposited at room temperature. It also has a much lower roughness than ITO. The high mobility of IZTO, which was comparable with ITO, was attributed to poor phonon scattering because of the amorphous structures.

In this study, we focus on fabricating IZTO films using DC magnetron sputtering powers by modifying the surface of ITO as a new transparent electrode. To test the effect of IZTO films as an electrode in ECD, we have fabricated an electrochromic device (ECD) consisting of a working electrode (WO_3_ electrode film deposited on IZTO/ITO/glass) and a counter-electrode (Pt mesh) in 0.2 M LiClO_4_/ PC solution.

## 2. Experimental

IZTO films (Sample 1−5) with thickness of approximately 250 nm were deposited on non-alkali glass substrates using DC magnetron sputtering technology. IZTO films were fabricated at a room temperature process with an IZTO target under various DC magnetron sputtering powers by increasing from 50 W to 150 W. The IZTO (70 at.% In_2_O_3_ + 10 at.% SnO_2_ + 20 at.% ZnO) target was grown in 99.99% purity with a diameter of 76.2 mm and a 3 mm thickness. The base chamber evacuated with a high-vacuum pressure set to less than 9.33 × 10^−4^ Pa. The working pressure, set at 0.13 Pa, was kept with an argon (Ar) gas flow of 30 sccm. Prior to deposition, the target was pre-sputtered for 3 min and the distance between the target and substrate was set at approximately 90 mm. The DC magnetron sputtering powers were varied from 50 W to 150 W. The deposition process of IZTO films are listed in Table 1. With the samples of the IZTO/ITO/glass substrate, we continued WO_3_ electrode film (220 nm) layer that was deposited by using cathodic arc plasma (CAP) deposition with a purity tungsten (W)-metal target (76 mm in diameter and 12 mm in thickness) at room temperature. The base chamber pressure was set to be less than 1.33 × 10^−3^ Pa using a turbo pump. In this study, we used an oxygen mass flow of 375 sccm and argon mass flow of 75 sccm for the reactive gases. The WO_3_ series were fabricated on IZTO/ITO/glass as electrochromic layers, which are listed in Table 2.

**Figure 2:**
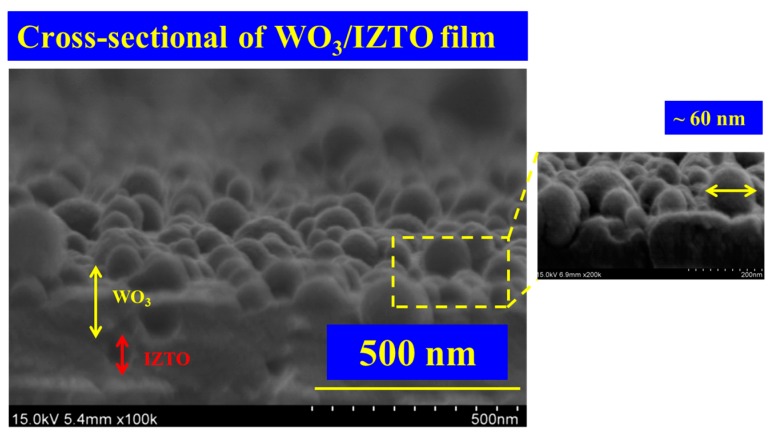
Cross-sectional of WO_3_/IZTO film.

We injected/extracted lithium ions (Li^+^ ions) into the WO_3_ film (electrochromic layer) by applying a negative/positive voltage. In the electrolyte system, we used liquid electrolyte composed of lithium perchlorate (LiClO4, M_w_ = 106.39, Sigma-Aldrich, Darmstadt, Germany) and propylene carbonate (PC, C_4_H_6_O_3_, Sigma-Aldrich), the resulting weight ratio was 0.053 (LiClO_4_/PC = 10.6 g/200 mL). An active area of 2 × 3 cm^2^ was used in our case for WO_3_/IZTO/ITO films. Figure 2 shows the cross-sectional of the WO_3_ nanoparticle electrode-modified IZTO thin films by CAP deposition used with O_2_/Ar = 5.

Crystallographic structures and phases of IZTO films were analyzed using glazing angle X-ray diffraction (XRD). The morphology of the IZTO film was analyzed through a scanning electron microscope (SEM). The electrical properties were characterized using a Hall effect measurement system at room temperature. A cycle voltammetry (CV) measurement was performed in order to understand the electrochemical properties of the WO_3_ electrode film deposited on an IZTO/ITO/glass substrate. The optical transmittance modulation for ECD was measured using a UV-Vis spectrometer (USB 4000, Ocean Optics, Inc., 830 Douglas Ave., Dunedin, FL, USA).

## 3. Results and Discussion

### 3.1. Basic Properties of IZTO Films with Various Sputtering Powers

**Figure 3:**
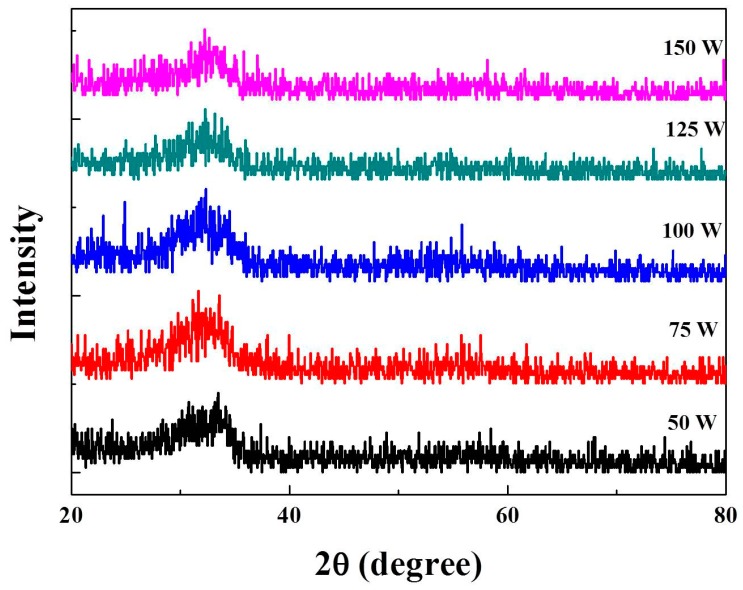
XRD of the IZTO films deposited with various sputtering powers.

Figure 3 shows the XRD patterns for IZTO films deposited on glass under various DC magnetron sputtering powers increasing from 50 W to 150 W. The XRD patterns exhibited broad peaks at a 2θ of 33°, and without other discernible, indicating amorphous structures of all samples regardless of the sputtering power. In a previously reported investigation of relative IZTO films, Zn was doped into ITO and the structure changed from crystalline ITO to amorphous In(ZnSn)O and In_2_Zn_5_O [9]. In general, ZnO-doped In_2_O_3_ could keep a stable amorphous structure below 500 °C [10]. On the other hand, the crystalline temperature of In_2_Zn_5_O_8_ was higher than 800 °C [11]. In our reports, relative IZTO films were shown to be fabricated at room temperature, which exhibited a stable amorphous structure.

**Figure 4:**
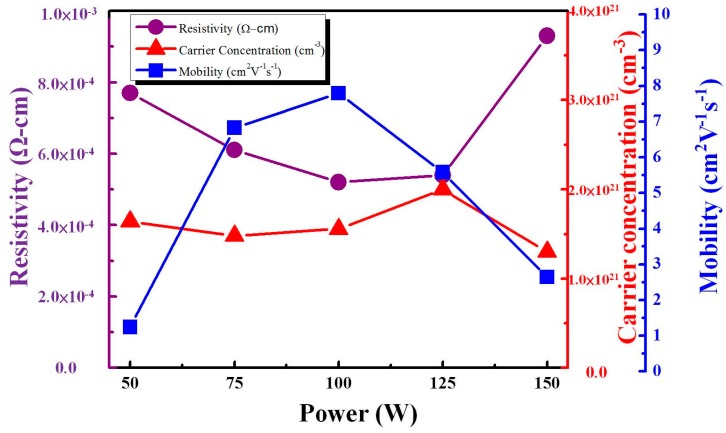
Electrical resistivity, carrier concentration, and mobility of IZTO films with various sputtering powers.

The electrical resistivity, carrier concentration, and mobility with various sputtering powers are shown in Figure 4. In Figure 4, it is shown that sputtering power increased from 50 W to 100 W, and both carrier concentration and carrier mobility increased, resulting in a decrease of electrical resistivity. The electrical resistivity reached the minimum value of 5.2 × 10^−4^ Ω·cm and the carrier mobility reached the maximum value of 7.93 cm^2^/V·s at 100 W. In our experiments, the carrier concentration varied from 1.64 × 10^21^ to 2.1 × 10^21^ cm^−3^ as the power was increased from 50 W to 150 W. Carrier concentration observed a maximum value of 2.1 × 10^21^ cm^−3^ at 125 W.

**Figure 5:**
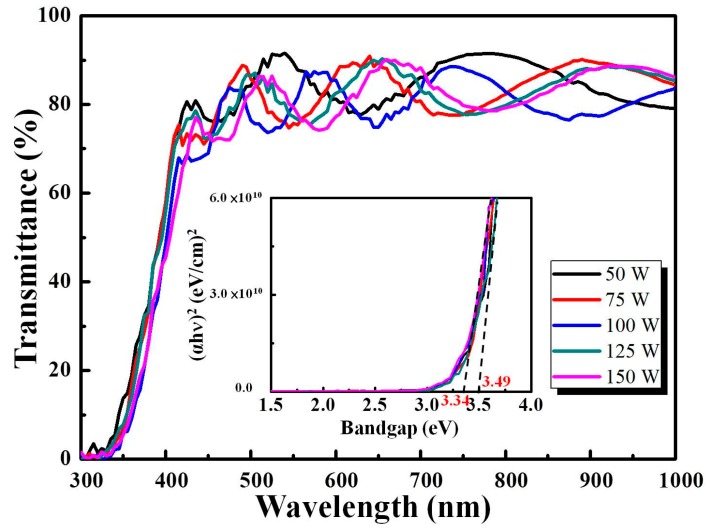
The optical transmittance spectra and band gap of IZTO films with different sputtering powers.

Figure 5 shows the optical transmittance spectra of an IZTO film with different sputtering powers increased from 50 W to 100 W. The average transmittance of an IZTO/glass film of 80.4% was achieved in a wavelength region of 400 nm–800 nm at 125 W, with a sharp absorption edge at approximately 330 nm. As shown in Figure 5, the optical band gap energy Eg shifted in the range from 3.34 eV to 3.49 eV. The Eg of an IZTO film was calculated by considering the electron transition from the valence to conduction band due to the absorbed photon energy hν. In a direct band gap, the absorption coefficient obeys the following relationship for Eg [12]:

(1) $$\;{\left(\alpha h\nu \right)}^{2}=h\nu -E_{g}\;$$

where *h* is Plank’s constant, and *v* is the photon frequency. The band gap is plotted by extrapolating the linear portion of ${\left(\alpha h\nu \right)}^{2}$ against the *hv* axis. In accordance to the Burstein–Moss effect [13], absorption edge blue-shift behavior was in accordance with increased electron concentration. The average transmittance of IZTO/glass film in a wavelength region of 400 nm–800 nm and band gap with different sputtering powers are listed in Table 3.

**Figure 6:**
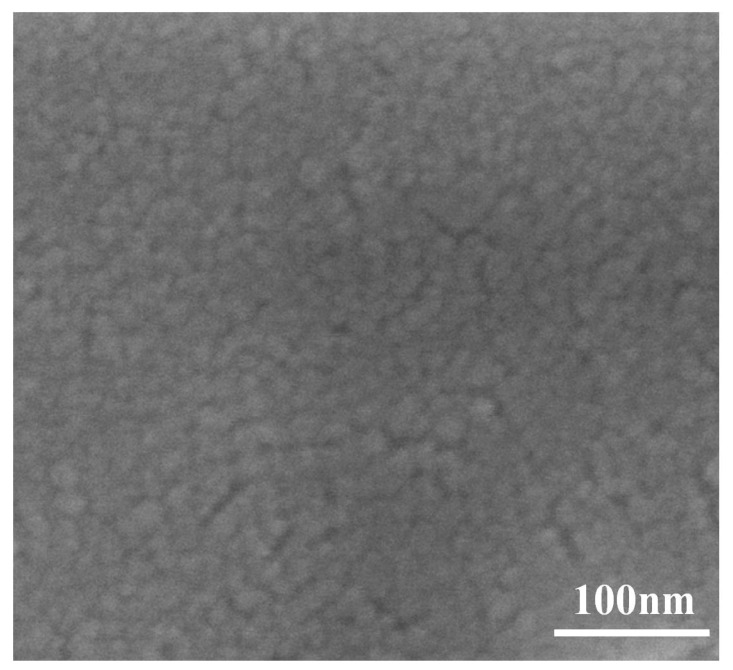
SEM morphology of the the IZTO film deposited at 100 W.

Figure 6 shows the surface morphology of the IZTO film deposited at 100 W. In Figure 6, the structure of the top view IZTO film was shown smooth without defects such as cracks and protrusions [14]. The smooth surface of the transparent conducting film is essential to accomplish high performance of optoelectronic devices because the surface roughness can short-circuit optoelectronic devices [15].

### 3.2. Properties of IZTO Films as Transparent Conducting Electrodes for Electrochromic Devices

To investigate the properties of IZTO films, a WO_3_ electrode film was deposited on IZTO/ITO/glass substrate. To understand the electrochemical properties of a WO_3_ electrode film deposited on IZTO/ITO/glass substrate, CV measurements were performed by constructing three electrode cells consisting of a working electrode (WO_3_ electrodes film deposited on IZTO/ITO/glass) and a counter-electrode (Pt mesh) in 0.2 M LiClO_4_/PC solution, as shown Figure 1. The CV curve of the current density and voltage traces were recorded within a linear potential sweep between −1.5 V and 0.5 V at a fixed scan rate of 150 mV/s. The coloration/bleaching electrochromic processes involved the injection/extraction (intercalation/deintercalation) of Li^+^ ions into/out of the WO_3_ electrode film following electrochemical reaction:

WO_3_ (bleaching) + *x* (Li^+^ + e^−^) → Li*_x_*WO_3_ (coloration) (2)

**Figure 7:**
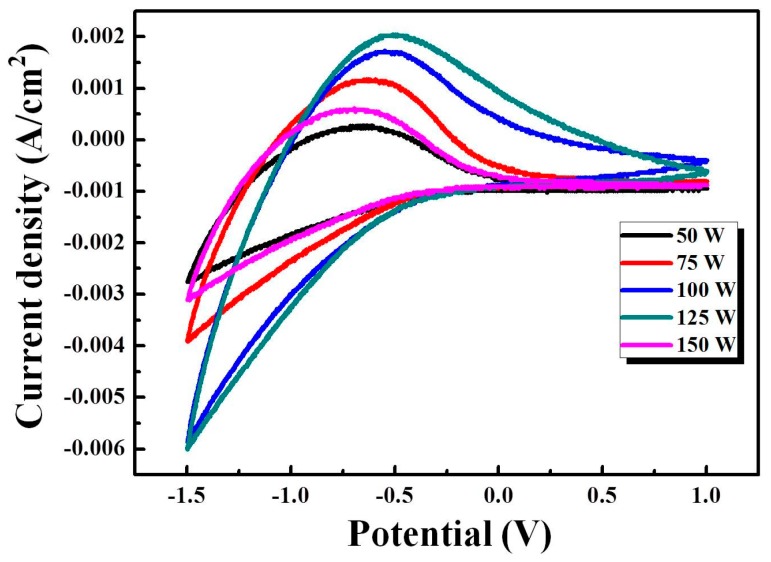
The 50th cycle CV curves of key factor IZTO films with various sputtering powers.

**Figure 8:**
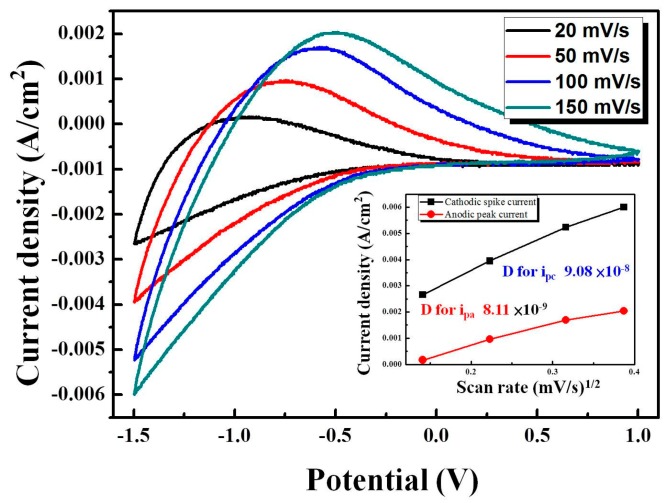
The CV curves of the 50th cycle of WO_3_/IZTO films at 125 W for various scan rates ranging from 20 to 150 mVs^−1^.

**Figure 9:**
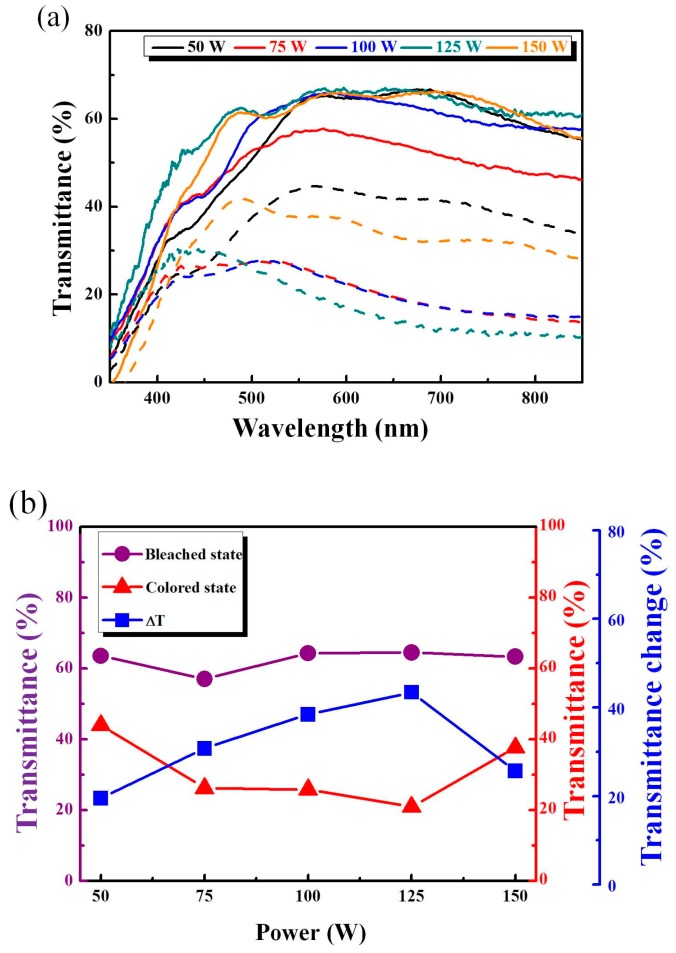
(**a**) The bleaching (solid line) and coloration (dotted line) state transmittance, and (**b**) bleaching, coloration, and ΔT = (T_bleaching_ − T_coloration_) of WO_3_/IZTO/ITO films at different sputtering powers.

During the cathodic scan, the reduction of W-ions W^6+^ to W^5+^ led to coloring state, and the reverse anodic scan (forward scan) the oxidation of W^5+^ to W^6+^ caused a bleaching phenomenon. When applying the negative voltage on the WO_3_ electrode film, the electrons and Li^+^ ions were inserted into the WO_3_ crystal structure, and W^6+^ reduced to a lower valence state W^5+^, as shown by the tungsten bronze structure Li*_x_*WO_3_ (coloration state) [16]. The 50th-cycle CV curves of key factor WO_3_/IZTO/ITO films with various powers (50 W, 75 W, 100 W, 125 W, and 150 W) are shown in Figure 7. The diffusion coefficients of Li^+^ ions in WO_3_/IZTO/ITO films could be evaluated by measuring the CV curves, and diffusion coefficients were a representative parameter to evaluate the structural properties of the films. The relationship between the peak current and the scan rate could be determined with the Randles–Servick equation for relating ion diffusion coefficients [17] as follows:

(3) $$\;J_{P}=2.69\times 10^{5}n^{3/2}C_{0}D^{1/2}\nu^{1/2}\;$$

where *C*_0_ is the concentration of the active ions in the electrolyte solution in mol·cm^−3^; *v* is the potential scan rate mVs^−1^; *D* is the diffusion coefficient in cm^2^s^−1^, *J_p_* is the peak current density in unit of area (working area 6 cm^2^), which includes *J_pc_* and *J_pa_* with oxidation and reduction of peak current density; and *n* is the number of electrons participated in the chemical reaction. The *J_pc_*, *J_pa_*, and the diffusion coefficient (*D)* are shown in Table 4. From Table 4, the resulting higher diffusion coefficients indicate a larger contact area and porosity with fast ion insertion/extraction. We observed the highest oxidation/reduction ion diffusion coefficients (9.38 × 10^−^^9^ and 8.12 × 10^−^^8^ cm^2^/s, respectively) with WO_3_/IZTO/ITO films at 125 W, indicating enhanced electrochromic properties compared to the other samples. Figure 8 shows that as scan rate increased, the anodic peak position moved to a higher potential position and peak current densities also increased at different scan rates that can be controlled by the diffusion coefficient of ions. The larger linear nature between *j_p_* and *v*^1/2^ of WO_3_/IZTO films are shown in the inset of Figure 8. This indicates that the reaction was fast and the insertion and extraction of ions were a diffusion-controlled process. In our case, for WO_3_/IZTO/ITO films with an active area *A* of 2 × 3 cm^2^ and *C*_0_ = 0.2 mol·cm^−3^, the Li^+^ ion diffusion coefficients of oxidation/reduction (8.11 × 10^−^^9^ and 9.08 × 10^−^^8^ cm^2^/s, respectively) were calculated from the slope of the current density versus potential curves. The area of the CV curves is deeply related to the charge stored (capacity) in the electrochemical process at the surface of WO_3_/IZTO films [18]. With CV curves having a larger cycle area, this indicates that more charges are taking part in redox reactions. In our results, a larger optical modulation was achieved during a larger enveloped area in the CV curve and larger carrier concentration. In Figure 9a, the optical transmittance spectra of WO_3_/IZTO films were controlled between bleaching and coloration plotted as a function of wavelength for IZTO films deposited at different sputtering powers. Furthermore, transmittance optical modulation (ΔT = T_bleaching_ − T_coloration_) for all samples are shown in Figure 9b. The optical transmittance changes of all sample increased from 20% to 44% as a function of sputtering powers. The transmittance optical modulation, ΔT = 44%, with 125 W was higher than the other samples at a fixed wavelength of 550 nm. The transmittance modulation increased due to a larger enveloped area in the CV curve and a larger carrier concentration.

**Figure 10:**
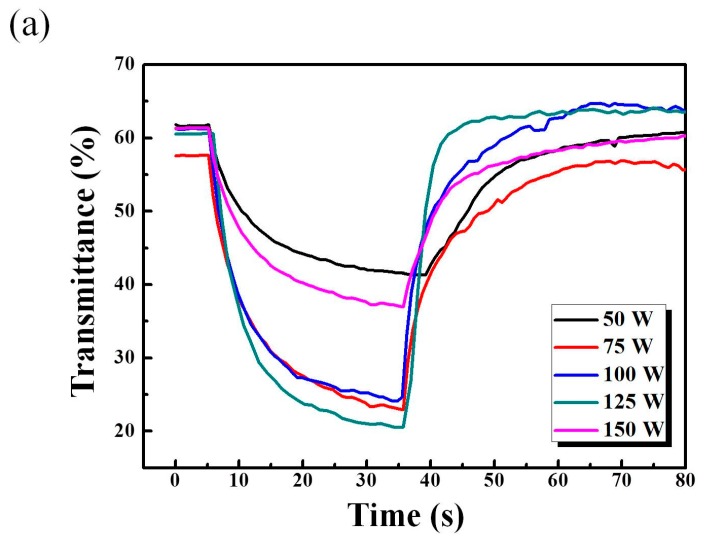

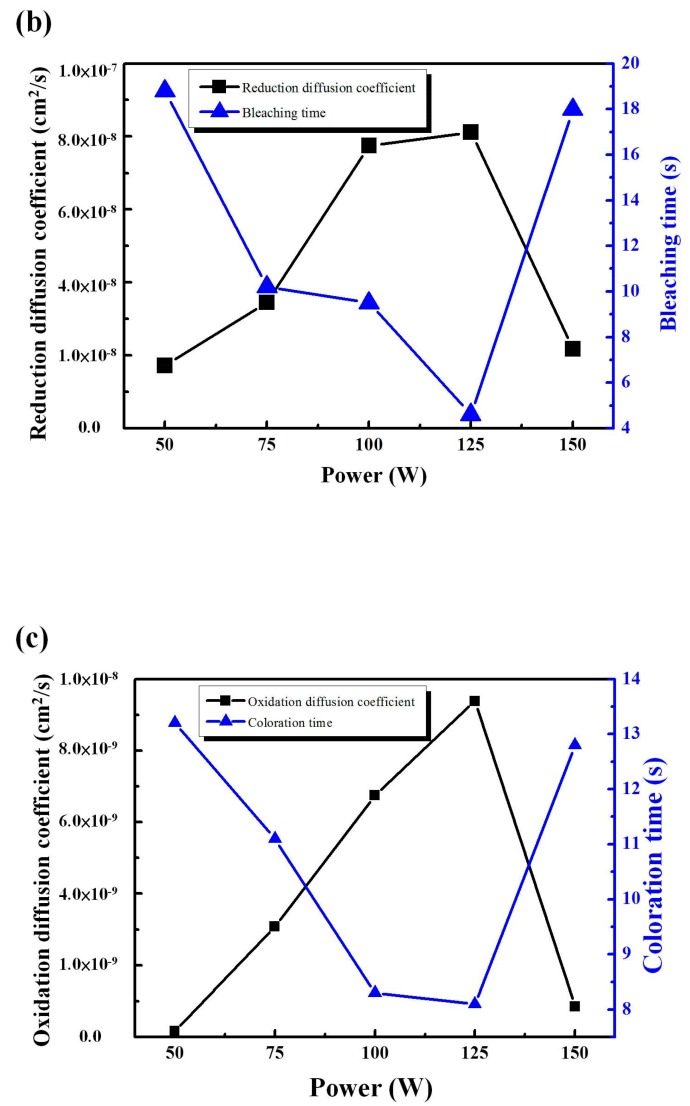
(**a**) Switching response time for coloration and bleaching states of WO_3_/IZTO films at a fixed wavelength 550 nm. (**b**) Bleaching switching time and reduction of ion diffusion coefficients. (**c**) Coloration switching time and oxidation of ion diffusion coefficients for WO_3_/IZTO films at different sputtering powers.

Figure 10 displays in situ transmittance of the switching response time of WO_3_/IZTO/ITO film samples prepared with various sputtering power tested with a pulsed potential between −1.3 V and 0.4 V. The switching response time was defined as the in situ time to reach 80% of the full change in transmittance between the bleaching and coloration states. The switching responses time, including the coloration (t_c_) and bleaching (t_b_) times, are shown in Figure 10b,c. The fast switching response times for sample 4 (at 125 W) was shown in Figure 8b,c. For sample 4 (at 125 W), the switching response times were 8.1 s from the bleached state to the colored state and 4.6 s during the reverse process due to higher diffusion coefficients indicating a larger contact area and porosity.

## 4. Conclusions

The structure and electrical properties of IZTO films prepared under various DC magnetron sputtering powers varying from 50 W to 150 W were investigated. We found that IZTO films had amorphous structures in all samples regardless of sputtering powers. The carrier concentration reached the maximum value of 2.1 × 10^21^ cm^−3^ at 125 W. We observed a higher ion diffusion coefficient, *D* = 8.12 × 10^−^^8^ cm^2^/s, for the IZTO film at 125 W, causing an optical contrast of 43%, and colored and bleached switching times were 8.1 s and 4.6 s at a wavelength of 550 nm.

## Figures and Tables

**Table 1:** Detail of parameters of the IZTO films with various sputtering powers.

Processing	Working Pres. (Pa)	Base Pres. (mPa)	Ar (sccm)	DC Power (W)	Thickness (nm)	Time (min)	Deposition Temp. (°C)	Deposition Rate. (nm/min)
sample-1	0.13	9.33 × 10^−4^	30	50	250	14.25	RT	17.54
sample-2	0.13	9.33 × 10^−4^	30	75	250	11	RT	22.72
sample-3	0.13	9.33 × 10^−4^	30	100	250	9.1	RT	27.47
sample-4	0.13	9.33 × 10^−4^	30	125	250	6.0	RT	41.66
sample-5	0.13	9.33 × 10^−4^	30	150	250	4.9	RT	51.02

**Table 2:** Deposition parameters of the WO_3_ electrode film.

Target	Working Pres. (Pa)	Base Pres. (mPa)	Ar/O_2_ (sccm)	Power (W)	Thickness (nm)	Time (min)	Deposition Temp. (°C)	Deposition Rate. (nm/min)
Metal W	2.7	1.3 × 10^−3^	75/375	1350	220	15	RT	14.67

**Table 3:** IZTO film with different sputtering powers based on average transmittance in a wavelength region of 400 nm–800 nm and the associated band gaps.

Sputtering Powers (W)	50 W	75 W	100 W	125 W	150 W
Average transmittance in a wavelength region (%)	83.64	80.47	79.42	80.41	79.11
Bandgap (eV)	3.37	3.37	3.39	3.49	3.34

**Table 4:** The diffusion coefficients of WO3/IZTO films at different sputtering powers.

Sample	IZTO (W)	Anodic Peak Current (*j_pa_*)	Cathodic Spike Current (*j_pc_*)	Diffusion Coefficient (cm^2^/s)	
				D for *i_pa_*	D for *i_pc_*
1	50	2.67 × 10^−4^	2.76 × 10^−^^3^	1.60 × 10^−^^10^	1.72 × 10^−^^10^
2	75	1.17 × 10^−4^	3.91 × 10^−^^3^	3.08 × 10^−^^10^	3.45 × 10^−^^10^
3	100	1.73 × 10^−^^3^	5.86 × 10^−^^3^	6.75 × 10^−^^10^	7.75 × 10^−^^10^
4	125	2.04 × 10^−^^3^	6.00 × 10^−^^3^	9.38 × 10^−^^10^	8.12 × 10^−^^10^
5	150	6.15 × 10^−^^3^	3.11 × 10^−^^3^	8.53 × 10^−10^	2.18 × 10^−^^10^

## References

[1] Granqvist C.G. (2006). Electrochromic materials: Out of a niche. Nat. Mater..

[2] Granqvist C.G. (2000). Electrochromic tungsten oxide lms: Review of progress 1993–1998. Sol. Energy Mater. Sol. Cells.

[3] Niklasson G.A., Granqvist C.G. (2007). Electrochromics for smart windows: Thin films of tungsten oxide and nickel oxide, and devices based on these. J. Mater. Chem..

[4] Jiao Z., Wang J., Ke L., Liu X., Demir H.V., Yang M.F., Sun X.W. (2012). Electrochromic properties of nanostructured tungsten trioxide (hydrate) films and their applications in a complementary electrochromic device. Electrochim. Acta.

[5] Wu W.F., Chiou B.S. (1994). Properties of radio-frequency magnetron sputtered ITO films without in-situ substrate heating and post-deposition annealing. Thin Solid Films.

[6] Sun X.W., Wang J.X. (2008). Fast Switching Electrochromic Display Using a Viologen-Modified ZnO Nanowire Array Electrode. Nano Lett..

[7] Kateb M., Safarian S., Kolahdouz M., Fathipour M., Ahamdi V. (2016). ZnO-PEDOT core-shell nanowires: An ultrafast, high contrast and transparent electrochromic display. Sol. Energy Mater. Sol. Cells.

[8] Liang L., Zhang J., Zhou Y., Xie J., Zhang X., Guan M., Pan B., Xie Y. (2013). High-performance flexible electrochromic device based on facile semiconductor-to- metal transition realized by WO_3_·2H_2_O ultrathin nanosheets. Sci. Rep..

[9] Moriga T., Edwards D.D., Mason T.O., Palmer G.B., Poeppelmeier K.R., Schindler J.L., Kannewurf C.R., Nakabayashi I. (1998). Phase relationships and physical properties of homologous compounds in the zinc oxide-indium oxide system. J. Am. Ceram. Soc..

[10] Ko Y.D., Kim Y.S. (2012). Room temperature deposition of IZTO transparent anode films for organic light-emitting diodes. Mater. Res. Bull..

[11] Ohashi N., Sakaguchi I., Hishita S., Adachi Y., Hareda H., Ogino T. (2002). Crystallinity of In2O_3_(ZnO)_5_ films by epitaxial growth with a self-buffer-layer. J. Appl. Phys..

[12] Serpone N., Lawless D., Khairutdinov R. (1995). Size Effects on the Photophysical Properties of Colloidal Anatase TiO_2_ Particles: Size Quantization versus Direct Transitions in This Indirect Semiconductor?. J. Phys. Chem..

[13] Chen W.L., Shen G.S., Wu Z., Li Z., Hong R.J. (2016). Optimizing transparent conductive Al-doped ZnO thin films for SiN_x_ free crystalline Si solar cells. J. Mater. Sci. Mater. Electron..

[14] Bae J.H., Moon J.M., Jeong S.W., Kim J.J., Kang J.W., Kim D.G., Kim J.K., Park J.W., Kim H.K. (2008). Transparent Conducting Indium Zinc Tin Oxide Anode for Highly Efficient Phosphorescent Organic Light Emitting Diodes. J. Electrochem. Soc..

[15] Tak Y.H., Kim K.B., Park H.G., Lee K.H., Lee J.R. (2002). Criteria for ITO (indium–tin-oxide) thin film as the bottom electrode of an organic light emitting diode. Thin Solid Films.

[16] Faughnan B.W., Crandall R.S., Heyman P.M. (1975). Electrochromism in tungsten(VI) oxide amorphous films. RCA Rev..

[17] Habib M.A., Glueck D. (1989). The electrochromic properties of chemically deposited tungsten oxide films. Sol. Energy Mater..

[18] Cai G., Darmawan P., Cui M., Wang J., Chen J., Magdassi S., Lee P.S. (2016). Highly Stable Transparent Conductive Silver Grid/PEDOT:PSS Electrodes for Integrated Bifunctional Flexible Electrochromic Supercapacitors. Adv. Energy Mater..

